# Dissection of the pulmonary artery: a rare complication

**DOI:** 10.36416/1806-3756/e2025-0362

**Published:** 2026-03-05

**Authors:** José Ricardo Bandeira de Oliveira, José Fabrício Macedo, Angela Maria Pontes Bandeira de Oliveira

**Affiliations:** 1. Instituto de Medicina Integral Professor Fernando Figueira, Recife (PE), Brasil.; 2. Real Hospital Português de Beneficência, Recife (PE), Brasil.; 3. Pronto-Socorro Cardiológico de Pernambuco, Recife (PE), Brasil.

**Figure 1:**
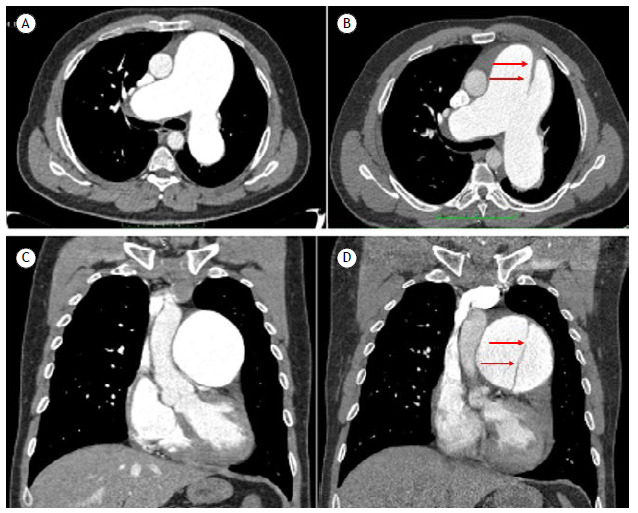
Initial chest computed tomography (CT) images showing aneurysmal dilatation of the pulmonary artery trunk and its main branches in axial (A) and coronal (B) views. On the right, follow-up CT images obtained six months later revealing a dissection flap in the pulmonary artery trunk (arrows in C and D).

A 38-year-old man with idiopathic pulmonary arterial hypertension was admitted with severe chest pain and worsening dyspnea. He was taking sildenafil (120 mg/day) and bosentan (250 mg/day). Initial laboratory tests were unremarkable, whereas electrocardiography revealed right ventricular (RV) overload. Chest computed angiotomography showed dissection of the pulmonary artery trunk (Figures 1C and 1D), which developed six months after the initial scan (Figures 1A and 1B). Echocardiography confirmed this finding, showing an intimal flap 3 cm from the pulmonary valve annulus. Mean pulmonary artery pressure was estimated at 70 mmHg, with signs of RV dysfunction. Despite supportive care, the patient developed recurrent chest pain, followed by cardiorespiratory arrest and sudden death.

Pulmonary artery dissection (PAD) is a rare and often fatal complication that typically occurs in patients with established pulmonary hypertension. It is more frequently observed in women, with a female-to-male ratio of 1.2:1, and the mean age at diagnosis is approximately 40 years. The clinical presentation is nonspecific, most commonly including acute chest pain, dyspnea, and cyanosis. In about 72% of cases, PAD involves the pulmonary artery trunk. Pathophysiologically, the initiating event is a tear between the intimal and medial layers of the vessel wall, leading to longitudinal dissection and the formation of a false lumen, which can precipitate catastrophic hemodynamic compromise.^1^ ^-^ ^3^
